# Presence of infectious agents and co-infections in diarrheic dogs determined with a real-time polymerase chain reaction-based panel

**DOI:** 10.1186/1746-6148-10-23

**Published:** 2014-01-16

**Authors:** Aline Baumann da Rocha Gizzi, Simone Tostes Oliveira, Christian M Leutenegger, Marko Estrada, Denise Adamczyk Kozemjakin, Rafael Stedile, Mary Marcondes, Alexander Welker Biondo

**Affiliations:** 1 Department of Veterinary Medicine, Federal University of Parana, 1540 R dos Funcionários, Curitiba, PR 80035-050, Brazil; 2 IDEXX Laboratories Inc., 2825 Kovr Drive, West Sacramento, CA 95605, USA; 3 Clinilab Laboratory of Animal Pathology, 894 R Holanda, Curitiba, PR 82540-040, Brazil; 4 Department of Animal Medicine, Federal University of Rio Grande do Sul, 9090 Avenue Bento Gonçalves, Porto Alegre, RS 91540-000, Brazil; 5 Department of Clinics, Surgery and Animal Reproduction, College of Veterinary Medicine, São Paulo State University, 793 R Clovis Pestana, Araçatuba, SP 16050-680, Brazil; 6 Department of Veterinary Pathobiology, University of Illinois, 2001 S Lincoln Avenue, Urbana, IL 61802, USA

**Keywords:** Canine, Co-infection, Diarrhea, Panel, Real-time PCR

## Abstract

**Background:**

Infectious diarrhea can be caused by bacteria, viruses, or protozoan organisms, or a combination of these. The identification of co-infections in dogs is important to determine the prognosis and to plan strategies for their treatment and prophylaxis. Although many pathogens have been individually detected with real-time polymerase chain reaction (PCR), a comprehensive panel of agents that cause diarrhea in privately owned dogs has not yet been established. The objective of this study was to use a real-time PCR diarrhea panel to survey the frequencies of pathogens and co-infections in owned dogs attended in a veterinary hospital with and without diarrhea, as well the frequency in different countries. Feces samples were tested for canine distemper virus, canine coronavirus, canine parvovirus type 2 (CPV-2), *Clostridium perfringens* alpha toxin (CPA), *Cryptosporidium* spp., *Giardia* spp., and *Salmonella* spp. using molecular techniques.

**Results:**

In total, 104 diarrheic and 43 control dogs that were presented consecutively at a major private veterinary hospital were included in the study. Overall, 71/104 (68.3%) dogs with diarrhea were positive for at least one pathogen: a single infection in 39/71 dogs (54.9%) and co-infections in 32/71 dogs (45.1%), including 21/32 dogs (65.6%) with dual, 5/32 (15.6%) with triple, and 6/32 (18.8%) with quadruple infections. In the control group, 13/43 (30.2%) dogs were positive, all with single infections only. The most prevalent pathogens in the diarrheic dogs were CPA (40/104 dogs, 38.5%), CPV-2 (36/104 dogs, 34.6%), and *Giardia* spp. (14/104 dogs, 13.5%). CPV-2 was the most prevalent pathogen in the dual co-infections, associated with CPA, *Cryptosporidium* spp., or *Giardia* spp. No statistical difference (P = 0.8374) was observed in the duration of diarrhea or the number of deaths (P = 0.5722) in the presence or absence of single or co-infections.

**Conclusions:**

Diarrheic dogs showed a higher prevalence of pathogen infections than the controls. Whereas the healthy dogs had only single infections, about half the diarrheic dogs had co-infections. Therefore, multiple pathogens should be investigated in dogs presenting with diarrhea. The effects of multiple pathogens on the disease outcomes remain unclear because the rate of death and the duration of diarrhea did not seem to be affected by these factors.

## Background

Canine infectious diarrhea has been considered a challenge for veterinarians because of its pathogenic variability and the concurrent presence of viral, bacterial, and protozoan co-infections [1,2]. Whereas some pathogens remain on the mucosal surface and produce potent enterotoxins that can disrupt the fluid flux, others penetrate and replicate within intact epithelial cells, producing inflammatory damage and/or destroying the host cells, which are overlapping pathological processes [3]. However, many dogs harbor potential pathogens without any clinical signs, so the cause–effect relationships are far from clear.

Molecular tools have been used for the identification and diagnosis of infectious diseases, in addition to conventional culture techniques and antibody-based tools [4,5]. Among these molecular approaches, the use of real-time polymerase chain reaction (PCR) has greatly improved the sensitivity and sensibility of standard PCR assays of pathogens in canine fecal samples [6].

Therefore, although a number of pathogens have been individually detected with real-time PCR, including *Salmonella* spp. [6] and pathogenic *Escherichia coli* strains [7], a comprehensive panel of potentially diarrhea-causing pathogens in owned dogs is yet to be established. Therefore, the aim of this study was to investigate pathogenic co-infections in populations of diarrheic and control owned dogs using a real-time PCR analysis of a panel of diarrhea-causing agents.

## Results

### Real-time PCR

Potential enteropathogens were identified in 71/104 (68.3%) diarrheic samples in the present study, most of which contained multiples pathogens. Single infections were observed in 39/71 (54.9%) positive samples and co-infection in 32/71 (45.1%) possible samples. Dual, triple, and quadruple infections were observed among the co-infections (Table 1). The most prevalent agent involved in co-infections was canine parvovirus type 2 (CPV-2), and 21/36 (58.3%) of the diarrheic samples positive for CPV-2 were associated with others agents, most commonly with *Clostridium perfringens* alpha toxin (CPA), *Cryptosporidium* spp., and *Giardia* spp. Although 13/43 (30.2%) of the feces samples from the control dogs were positive for the panel of diarrhea-causing pathogens, no co-infection was observed in any of these samples.

**Table 1:** Prevalence of single or co-infection in diarrheic and control feces using a real-time PCR panel

	**Dogs**		**P value**
**Infection**	**Control (43) n (%)**	**Diarrheic (104) n (%)**	
Negative	30/43 (69.8)	33/104 (31.7)	< 0.0002
Positive	13/43 (30.2)	71/104 (68.3)	< 0.0002
Single	13/13 (100)	39/71 (54.9)	0.004
Co-infection	None	32/71 (45.1)	0.004
Dual	None	21/32 (65,6)	-------
Triple	None	5/32 (15.6)	-------
Quadruple	None	6/32 (18.8)	-------

Of the 71/104 (68.3%) dogs with diarrhea and positive results, 26.8%, 28.2%, and 4.2% had pure viral, bacterial, and protozoan infections, respectively, whereas 7.0%, 14.0%, and 9.3% of samples from the control dogs were positive for viral, bacterial, and protozoan infections, respectively. The association of viral and bacterial infections was the most prevalent type of co-infection in the diarrheic group (37.5%), with CPV-2 and canine coronavirus (CCoV) constituting 75.0% of this type of co-infection. Viral and protozoan co-infections accounted for 25.0% of these associations, bacterial and protozoan for 6.2%, and viral, bacterial, and protozoan co-infections for 21.9% (Table 2).

**Table 2:** Virus, bacteria and protozoan association in diarrheic and control feces of dogs

		**Dogs Brazil**	
**Pure infection**	**P value**	**Control (43) n (%)**	**Diarrheic (104) n (%)**
Viral	0.01306	3/43 (7.0)	19/71 (26.8)
Bacterial	0.10720	6/43 (14.0)	20/71 (28.2)
Protozoan	0.42284	4/43 (9.3)	3/71 (4.2)
**Co-infection**			
Viral and viral		None	3/32 (9.4)
Viral and bacterial		None	12/32 (37.5)
Viral and protozoan		None	8/32 (25.0)
Bacterial and protozoan		None	2/32 (6.2)
Viral, bacterial and protozoan		None	7/32 (21.9)

A significant association was found between dogs with diarrhea and positive results on the real-time PCR diarrhea panel compared with the control dogs with normal feces (P < 0.001).

The detection of individual pathogens in the panel with real-time PCR (Table 3) showed that CPA was the most prevalent pathogen in the fecal samples, infecting 40/104 (38.5%) diarrheic dogs and 6/43 (14.0%) control dogs, and the difference between the groups was highly statistically significant (P = 0.006). Despite this, it was necessary to quantify CPA to consider its probable role. When the pre-established cutoff of > 300,000 copies/g of feces was applied [8], a significant difference (P = 0.0025) between the control group (4/43, 9.3%) and the diarrheic group (37/104, 35.6%) was observed. CPV-2 was the second most prevalent pathogen, found in 36/104 (34.6%) diarrheic dogs and 0/43 (none) control dogs, and also showed a highly significant difference between the groups (P < 0.001).

**Table 3:** Individual infectious agents in the real-time PCR canine diarrhea panel of dogs from Southern Brazil

**Pathogen**	**(Diarrheic dogs)**		**(Control dogs)**		
	**No. positive (%)**	**95% CI**	**No. positive (%)**	**95% CI**	**P value**
	**(n = 104)**		**(n = 43)**		
Overall rate*	71 (68.3)	58.3- 76.9	13 (30.2)	17.67 - 46.3	< 0.001
Canine distemper virus**	9 (8.7)	4.3 - 16.2	0 (0)	0 – 10.2	0.058
Canine parvovirus type 2*	36 (34.6)	25.7 - 44.7	0 (0)	0 – 10.2	0.001
***Salmonella *** **spp.**	1 (1)	0.05 - 6.01	0 (0)	0 – 10.2	1
***Cryptosporidium *** **spp.**	8 (7.7)	3.6 - 15.0	2 (4.7)	0.8 - 17.1	0.723
**Giardia spp.**	14 (13.5)	7.8 - 21.9	2 (4.7)	0.8 - 17.1	0.151
**Canine coronavirus**	12 (11.5)	6.4 - 19.7	3 (7)	1.8- 20.1	0.554
***C. perfringens *** alpha toxin gene*	40 (38.5)	29.2 – 48.5	6 (14)	5.8 – 28.6	0.006

*Significantly (P < 0.05) different between Diarrhea Group and Control Group.

**Significantly (P < 0.1) different between Diarrhea Group and Control Group.

Although 9/104 (8.7%) diarrheic dogs were positive for canine distemper virus (CDV), the difference between the diarrheic and control dogs was not significant (P = 0.059). Similarly, 12/104 (11.5%) diarrheic and 3/43 (7.0%) control dogs were positive for CCoV, and these rates were not significantly different (P = 0.554).

Only 1/104 (1.0%) and 8/104 (7.7%) diarrheic dogs were positive for *Salmonella* spp. and *Cryptosporidium* spp., respectively. In the control group only 2/43 (4.7%) were positive for *Cryptosporidium* spp., and the two groups did not differ significantly (P = 0.724). Although *Giardia* spp. were detected in 14/104 (13.5%) diarrheic dogs and 2/43 (4.7%) control dogs, the difference was not significant (P = 0.151).

### Diarrhea in relation to age

Among the diarrheic samples, 43/104 were from 0–1-year-old dogs, 36/104 from 1–8-year-old dogs, and 25/104 from dogs > 8 years old. The PCR results were positive in 39/43 (90.7%) samples from animals 0–1 years old, in 20/36 (55.5%) samples from those 1–8 years old, and 12/25 (48.0%) samples from those > 8 years old. A significant association was observed between dog age and the pathogens CPV-2 (P < 0.0001), *Giardia* spp. (P = 0.01), and CCoV (P = 0.02), which were most prevalent among the 0–1-year-old dogs. Although CPV-2 is considered to be primarily a disease of puppies, it also occurred in 4/36 (11.1%) animals aged 1–8 years and in 3/25 (12.0%) dogs aged > 8 years. Only 13/36 (36.1%) of the positive animals for CPV-2 had no history of vaccination, in which 1/4 (25%) and 1/3 (33.3%) were adults with 1-8 years and 8 years respectively. Details of dates and brands of the vaccines were not obtained. There was a highly significant association between age and co-infection (P < 0.0001), which was more prevalent among the 0–1-year-old dogs, of which 25/43 (58.1%) had co-infections with two or more pathogens. Co-infection occurred less often in the other age groups, occurring in 5/36 (13.8%) dogs in the 1–8-year-old group and 3/25 (12%) dogs > 8 years old. A significantly higher prevalence (P = 0.0026) of viral and protozoan associations was observed in the 0–1-year-old diarrheic dogs than in the other age groups. However, no significant associations between the other co-infections and age were observed.

### Diarrhea duration in relation to number of pathogens

No significant association (P = 0.8374) was observed between the duration of diarrhea and the presence of single or co-infections.

### Diarrhea in relation to death

The presence of co-infection did not increase the number of deaths (P = 0.5722) more than the presence of a single infection.

### Diarrhea in relation to clinical suspicion

In this study, even when diarrhea lasted for more than 10 days, an infectious disease was still clinically suspected (P = 0.6606).

### Diarrhea in relation to the use of antibiotics

During the period from sample collection to the generation of results for the diarrhea panel analysis (approximately five days), 40/104 (38.5%) dogs received empirical treatment with combinations of two to four antibiotics. In total, 40/71 (56.34%) diarrheic dogs with positive PCR results were treated with antibiotics that were, according to the literature, inappropriate for the pathogens ultimately identified.

### Parasitological diagnosis

In total, 20/104 (19.2%) and 3/43 (7.0%) fecal samples from diarrheic and control dogs, respectively, were positive on parasitological tests. The most prevalent intestinal parasites found in the diarrheic samples were protozoa (*Giardia* spp. and/or *Isospora* spp.), which affected 12/104 (11.5%) dogs, and helminths (*Ancylostoma* sp. and/or *Toxocara* sp.), which affected 10/104 (9.6%) dogs. All the parasites found in the three positive control samples were identified as *Ancylostoma* sp. When all the samples from diarrheic and control dogs were considered, 22/23 (95.7%) positive fecal samples and 61/124 (49.2%) negative fecal samples on parasitological tests were also positive on the real-time PCR diarrhea panel.

Of the 13/147 (8.8%) samples positive for helminths (*Ancylostoma* sp. and/or *Toxocara* sp.), 12/13 (92.3%) were also positive on the real-time PCR. Among the 134/147 (91.2%) samples negative for helminths, 71/134 (52.9%) were positive on the real-time PCR. Dogs positive for helminths were 1.7 times more likely to be positive on the real-time PCR diarrhea panel, which indicates a statistically significant association (P = 0.006) between the infections detected with real-time PCR and the presence of helminths.

Of the 16/147 (10.9%) samples shown to be positive for *Giardia* spp. with real-time PCR, only five were positive on the parasitological tests. Considering that real-time PCR is the gold standard for the detection of pathogenic agents, the parasitological test showed 31.2% sensitivity (95% confidence interval, 12.1%–58.5%). *Isospora* spp. were found in 7/147 (8.4%) samples with the parasitological test, and all of these samples were also positive for the agents detected with real-time PCR, suggesting that *Isospora* spp. are usually involved in co-infections.

### Results between countries

The prevalence of diarrheic dogs observed in the Brazilian samples (68.3%) was similar to that observed in the United States (54.5%), Australia (58.4%), Canada (52.0%), United Kingdom (51.7%), and Japan (49.6%), as shown in Table 4. The rate of co-infection observed in Brazilian diarrheic dogs (45.1%) was also higher than those in the other countries tested (Table 4) and a significant difference was observed between United States (P < 0.0001), Australia (P = 0.01) and Canada (P = 0.04).

**Table 4:** Canine diarrhea panel from diarrheic dogs of Brazil, United States, Australia Canada, UK and Japan

**Infectious agent**	**Brazil**	**US**	**Australia**	**Canada**	**UK**	**Japan**
	**Prevalence**					
Canine distemper virus	8.7%	1.4%***	2.3%**	1.6%***	2.5%**	1.0%***
*Salmonella* spp.	1.0%	2.2%	4.6%	5.0%	0.0%	3.0%
Canine parvovirus type 2	34.6%	1.9%***	6.8%***	8.0%***	35.1%	9.0%***
*Cryptosporidium* spp.	7.7%	5.4%	4.0%	14.0%	34.7%***	9.0%
*Giardia* spp.	13.5%	10.5%	10.8%	14.0%	20.2%	10.0%
Canine coronavirus	11.5%	13.9%	5.1%*	11.0%	16.9%	26.0%***
*C. perfringens* alpha toxin	38.5%	36.2%	45.6%	46.0%	51.2%*	21.0%***
**Overall infection rate**	68.3%	54.5%**	58.4%	52.0%**	51.7%**	49.6%***
**Coinfection rate**	45.1%	24.9%***	31.3%*	34.0%*	41.9%	35.3%
Samples included	n = 104	n = 7829	n = 526	n = 2855	n = 674	n = 486

*Significantly (P < 0.05).

**Significantly (P < 0.01).

***Significantly (P < 0.001).

## Discussion

A high prevalence (68.3%) of diarrheic dogs, shown to harbor at least one pathogen by real-time PCR, was observed in the Brazilian samples, which exceeded those in the United States (54.5%), Australia (58.4%), Canada (52.0%), United Kingdom (51.7%), and Japan (49.6%), as shown in Table 4. The rate of co-infection observed here in diarrheic dogs (45.1%) was also higher than those in the other countries tested. Despite the higher prevalence of enteropathogens and co-infections in Brazil, the rates in the other countries are also relevant, indicating that infectious diarrhea may be a global phenomenon rather than a phenomenon specific to a particular country. Because all dogs with co-infections belonged to the diarrheic group and co-infections were observed in all age categories, this study highlights the importance of investigating multipathogen co-infections, especially in dogs aged 0–1 years, in which the rate of co-infection was 4-fold higher than in the other age groups.

Many enteric viruses, bacterial pathogens, and parasites probably contribute to disease both individually and in combination [9], and together, co-infecting pathogens may cause more severe diarrhea than infections with each pathogen alone [10]. The pathogens involved in a co-infection can interact synergistically, for example via the host’s immune system, with the presence of one enhancing the abundance and/or virulence of the other, resulting in even greater pathogenesis and a greater contribution to the overall disease burden [11,12]. Therefore, interspecific pathogen interactions can alter the pathogen dynamics, host health, and the success of control strategies [13,14].

In this study, co-infection did not increase the duration of diarrhea and there was no significant difference in the number of deaths in animals with or without co-infections. Because there was no reliable correlation between the interaction of enteropathogens in co-infections in this study, the cause–effect relationship between the presence of an organism and the occurrence of diarrhea is still unclear. Opportunistic or commensal organisms may be identified from an imbalance in the intestinal flora or dysbiosis, and not all the co-infecting agents present must be treated to produce a good outcome. However, because all the infectious agents evaluated here have been described as causing diarrhea in experimental studies, knowledge of their presence allows treatments and prevention strategies to be planned. In this study, even when diarrhea persisted for more than 10 days, the infectious diseases were still present in the differential diagnosis. Empirical treatments and the use of several antibiotics are common in routine veterinary practice and the use of a panel to detect multiples pathogens prevents the incorrect or excessive use of antimicrobial drugs, which could cause resistance. Furthermore, some of these pathogens are potential zoonotic agents, including hookworms, *Giardia* spp., *Cryptosporidium* spp., and *Salmonella* spp., and the identification of these organisms can reduce the risk of their transmission to humans and others animals.

Although this study focused on client-owned dogs, dogs received in animal shelters are also expected to carry pathogen co-infections, including zoonotic agents [15]. However, differences have been observed in the prevalence of each agent, especially in terms of the co-infection rates, and dogs from shelters with diarrhea showed a higher prevalence of co-infection (96.0%) [15] than was observed in this study (45.1%). That heterogeneous dog population had a higher rate of crowding, and the dogs may have been immunocompromised for clinical, nutritional, and/or psychological reasons, exposed to more environmental pathogens, and sometimes with inadequate health care, so their high co-infection rate cannot be compared validly with that of owned dogs in households.

The highest rate of co-infection in this study involved the association of viral and bacterial agents, in contrast to the highest co-infection in dogs in the United States, which was caused by viruses and protozoans. The highest co-infection rates were for CPV-2 and CPA, observed in 9/12 (75.0%) samples from dogs in Brazil, and for CCoV and *Giardia* spp., which occurred together in 35.4% of dogs from the United States. These co-infections may have clinical effects and may require more-intensive efforts to ensure the appropriate treatments to eliminate specific pathogens and to correct electrolyte, acid–base, and nutritional disturbances, potential sepsis, and other metabolic consequences [16].

The alpha toxin gene is present in all strains of *Clostridium perfringens* and may be found in asymptomatic dogs as part of the normal intestinal microflora [17], as in 14% of the control dogs in the present study. Data from some studies indicate that conventional PCR that targets only CPA will almost always be positive and of virtually no clinical use [17]. However, a recent study demonstrated that the quantification of CPA may be used as a diagnostic marker for association of the agent in patients with diarrhea [8]. Using the same methodology and cutoff value as a previous study [8], we observed a significant difference between the control group (4/43, 9.3%) and in the diarrheic group (37/104, 35.6%; P = 0.0025) in the proportion of animals positive for > 300,000 copies of CPA. The higher amount of CPA in the diarrheic dogs than in the control dogs suggests that the high concentrations of toxins produced by this organism exert a pathogenic effect on the gastrointestinal tract. In the present study, diarrheic dogs co-infected with CPA had 3-fold more copies than those that were infected with only CPA. We hypothesize that in these cases, *C. perfringens* overgrowth in the bacterial flora increases the toxin expressed, which contributes to the dog’s diarrhea.

All the control dogs were negative for CPV-2, which was strongly associated with diarrhea (P = 0.000004), with an overall occurrence of 36/104 (34.6%) in the diarrheic dogs. The same prevalence (18/51, 34.6%) was observed in a study also conducted in Brazil but performed only with puppies up to 6 months old [18] and corroborated with previous surveys, which reported rates varying from 16% [19] to 58% [20]. Although CPV-2 has been considered to be primarily disease of puppies, the present study has shown the importance of also investigating adult dogs for CPV-2, since occurred in 11.1% of 1–8 year old dogs and in 12.0% of dogs older than 8 years. The CPV-2 was most prevalent agent involved in co-infections in this study, in which 58.3% of the diarrheic samples positive for CPV-2 were associated with others agents, contrasting with only 3.8% CPV-2 co-infection observed in the study with puppies [18]. This variability may be related to the geographic regions examined, the populations studied, agents investigated and the diagnostic techniques used [20]. Although the date of live-modified vaccination was not the focus of this study, the CPV-2 cutoff value used was able to differentiate vaccine strains from wild-type infections. Despite the use of vaccination, the CPV-2 was still spread among the dogs as observed in this study, in which only 13/36 (36.1%) of the positive animals for CPV-2 had no history of vaccination. Still remains unclear whether type-2 vaccines can provide protection against the new variants of the CPV, but a recent study observed that the cases of vaccine failure are most likely not associated to the mutations detected in the sequenced regions [21]. Thus, further studies should elucidate whether local parvovirus strains are effectively controlled by the currently available vaccines and which factors may be associated with the vaccination efficacy. CPV-2 was the most prevalent agent associated with dual co-infections in the present study, which may be attributable to highly contaminated environments or low dog immunity [22].

Although the detection rate of CCoV was higher in the diarrheic dogs (11.54%) than in the control dogs (6.98%), the lack of a statistically significant difference in these rates may indicate a secondary role for CCoV as an intestinal pathogen in dogs. Although the shedding of CPV-2 seemed to be associated with clinical signs of gastroenteritis, CCoV was also detected in healthy dogs, as previously reported [19]. Although CCoV infections are characterized by high morbidity and low mortality, with typically mild enteritis in dogs [23,24], 11/12 (91.7%) diarrheic dogs with CCoV were co-infected with other enteric pathogens, which may have aggravated their clinical signs and even caused higher mortality, as reported earlier for CPV-2 [25], canine adenovirus type 1 [26], and CDV [27]. CDV tended to be significantly higher in the diarrheic dogs than in the control dogs; although not significant, the P value was very close to the limit of significance (P = 0.059).

Whereas some enteropathogens, such as *Salmonella* spp., *Cryptosporidium* spp., and *Giardia* spp., showed similar prevalences in the various countries tested (Table 4), CDV and CPV-2 were approximately 4-fold more prevalent in Brazil, indicating that the higher incidence of viral diseases may be related to differences in strain pathogenicity, vaccination status, and environmental factors.

Although the CPV-2 strain and vaccine status/response may play important roles in viral infection and host immunity, further studies are required to fully understand this specific pattern of CPV-2 infections in Brazil.

Although intestinal parasites contribute to diarrhea, 95.7% of positive and 49.2% negative samples on the parasitological tests were also positive on the real-time PCR diarrhea panel, indicating that fecal parasitological tests alone should not be used as a single diagnostic tool.

## Conclusions

To the best of our knowledge, this is the first real-time PCR-based analysis of a panel of diarrhea-causing pathogens performed in Brazil. Our results indicate that dogs with diarrhea show a relatively high prevalence of pathogenic infections, highlighting the importance of investigating infectious pathogens in dogs with diarrhea. Whereas asymptomatic dogs had single infections only, approximately half the diarrheic dogs presented with co-infections, emphasizing the importance of the simultaneous investigation of multiple pathogens in individual samples. The most prevalent pathogens in diarrheic dogs in Brazil were *C. perfringens* alpha toxin, CPV-2, and *Giardia* spp. Among the dual co-infections observed, CPV-2 was most commonly associated with *C. perfringens* alpha toxin, *Cryptosporidium* spp., and *Giardia* spp.

In this study, co-infection did not increase the duration of diarrhea or increase the risk of death. However, considering that all the infectious agents examined have been described as causing diarrhea in experimental studies, knowledge of their presence should allow us to plan treatments and prevention strategies. The effects of multiple pathogens on the disease outcomes remain unclear, because neither the death rate nor the duration of diarrhea seemed to be affected by these factors.

## Methods

### Study population

A total of 147 (104 diarrheic and 43 asymptomatic) dogs consecutively presented by owners to the largest private veterinary hospital of Curitiba City, Southern Brazil, were included in the study for the realization of the real-time PCR diarrhea panel, parasitological diagnosis and clinical data collection.

Fresh fecal samples were collected from all 147 Brazilian dogs, placed in plastic vials, and kept refrigerated at 4°C until processing. At sampling, the fecal specimens were scored according to a five-point fecal scoring system for dogs, as previously described [28]: 1, liquid or watery feces with no form; 2, very soft, unformed feces; 3, very soft, moderately formed feces; 4, firm, well-shaped and cylindrical feces. Diarrheic dog feces included those with scores from 1 to 3, whereas the control dogs had no history of gastrointestinal disorders (vomiting, diarrhea, or anorexia) and fecal samples with a score of 4.

Additionally, results from 12,370 commercial samples using the same real-time PCR diarrhea panel performed in the Brazilian samples were obtained from samples originated from United States (7829), Australia (526), Canada (2855), United Kingdom (674) and Japan (486). The real-time PCR results for samples collected in different countries (Table 4) were obtained either by shipping the samples to the United States for commercial real-time PCR analysis (samples from Brazil, United States, Australia, United Kingdom, and Japan) or by allowing the diagnostic samples to be analyzed with the same real-time PCR procedure by the same service provider in the country of origin (Canada). Nucleic acid quantities, stability, and quality were assessed using internal sample controls when the samples were shipped to the United States for real-time PCR analysis.

The study was approved by the Ethics Committee in Animal Experimentation and Animal Welfare of the Federal University of Parana Curitiba Campus, Paraná State, Southern Brazil (protocol number 036/2011).

### Clinical outcomes

The diarrheic dogs were evaluated for the duration of diarrhea and death in relation to the presence of single or co-infections. Was also evaluated whether the clinical suspicion of association with infectious agents was related to the duration of diarrhea (≤ 10 days, 10 days to 2 months, and > 2 months) and whether the correct antibiotics were used (for empirical treatment after sample collection but before the results were obtained) based on the pathogen ultimately detected.

### Real-time PCR

Total nucleic acids were extracted with standard protocols using a commercial platform (Corbett XTractor-Gene, Qiagen, Valencia, CA, USA). A housekeeping gene (18S rRNA) was used to determine the amounts of genomic DNA and cDNA after reverse transcription and to confirm DNA integrity.

The PCR tests were based on the IDEXX proprietary RealPCR™ service platform (IDEXX Laboratories, Inc., Westbrook, ME, USA). Briefly, conserved nucleotide regions were selected and two primers and a hydrolysis probe were designed to hybridize to them, using commercial software (PrimerExpress Version 3.0, Applied Biosystems, Foster City, CA, USA). An additional two primers flanking the fragment amplified in the real-time PCR assay were designed for sequence verification purposes. Fecal samples were tested by hydrolysis probe-based real-time PCR assay for a panel of potential enteropathogens, including CDV (phosphoprotein G, GenBank acccession number AY649446.1), CCoV (M gene, Type I, AF502583; typ eII D13096), CPV-2 (VP2, U22139), CPA (alpha toxin, L43545), *Cryptosporidium* spp. (ssrRNA, A093489), *Giardia* spp. (ssrRNA, DQ836339), and *Salmonella* spp. (invasion A gene, EU348366) at a reference laboratory within 5 days after collection. CPA was quantified using a pre-established cutoff of > 300,000 copies/g of feces [8]. For CPV-2 a cutoff value was used to discriminate vaccine strains from wildtype infections (1.2 million CPV-2 gene equivalents per gram of stool). Real-time PCR was performed with standard primer and probe concentrations using a commercially available mastermix (LC480 ProbesMaster, Roche Applied Science, Indianapolis, IN, USA) on a commercially available real-time PCR platform (Roche LightCycler 480).

### Analytical and clinical validation

Real-time PCRs were validated analytically and clinically. For the analytical validation, each assay was required to meet six validation criteria: amplification efficiency, linearity, intra-run reproducibility, inter-run reproducibility, r^2^ value, and signal-to-noise ratio of the fluorescent signal using a specific synthetic positive control. Each assay had an analytical sensitivity of 10 molecules and an amplification efficiency between 95% and 100%. Clinical samples were selected based on a reference method for each test and a correlation study was performed. The analytical specificity of all real-time PCR tests was confirmed by resequencing the clinical sample material using additional primer pairs positioned outside the synthetic positive control. Diagnostic sensitivity and specificity based on comparisons with reference testing methods were in the high 90% for each real-time PCR test. Gold standard tests included the immunofluorescence antibody assay for CDV, enzyme-linked immunosorbent assay for CPV-2 and *Giardia*, and real-time PCR performed at the University of California Davis for CCoV, *Cryptosporidium*, and *Salmonella*.

To validate each PCR panel test result, seven quality controls were used for each sample tested: 1) PCR positive controls (quantitative); 2) PCR negative controls; 3) negative extraction controls (five per 96-well plate); 4) DNA internal sample control targeting the host 18S rRNA to quantify the DNA in the submitted diagnostic sample; 5) RNA internal sample control targeting the host 18S rRNA to quantify the cDNA in the submitted diagnostic sample after reverse transcription; 6) control to monitor environmental contamination; and 7) spike in the internal positive control to monitor PCR inhibition. These controls were used to assess the functionality of the PCR test protocol (1), the absence of contamination in the reagents (2) and laboratory (5), the absence of cross-contamination during the extraction process (3), the quality and integrity of the DNA and RNA as a measure of sample quality (4 and 5), the RT protocol (5), the absence of random positive PCR signals within the PCR laboratory (6), and the absence of PCR inhibitory substances carried over from the sample matrix (7).

### Parasitological diagnosis

A fecal flotation procedure was performed with zinc sulfate [29] and saturated sodium chloride [30] flotation solutions, as described previously. The parasitological test was performed with light microscopy by identifying the parasite eggs, larvae, cysts, and oocysts, according to their morphological characteristics [30,31].

### Statistical analysis

The results were subjected to statistical analysis with the χ^2^ test or Fisher’s exact probability. Differences were considered significant when *P* < 0.05. The statistical analysis was performed at a website for statistical computation (VassarStats, Vassar College, Poughkeepsie, NY, USA).

## Abbreviations

CDV: Canine distemper virus; CCoV: Canine coronavirus; CPV-2: Canine parvovirus type 2; CPA: *Clostridium perfringens* alpha toxin.

## Competing interests

The authors declare that they have no competing interests.

## Authors’ contributions

ABRG, STO, AWB, CL conceived the study, participated in its design and coordination. ABRG, DAK, CL, ME participated in data acquisition and performed the laboratory assays. RS conducted the statistical analyses. All authors participated in drafting the manuscript, read and approved the final manuscript.
